# Recent advances in tumor-targeting chemotherapy drugs

**DOI:** 10.20517/cdr.2021.86

**Published:** 2021-09-08

**Authors:** Iwao Ojima

**Affiliations:** Institute of Chemical Biology and Drug Discovery, Stony Brook University, Stony Brook, NY 11794-3400, USA.

The first antibody-drug conjugate (ADC), Mylotarg, received FDA approval in 2000, and the revolutionary targeted cancer chemotherapy drug, Imatinib (Gleevec), was approved by FDA in 2001. Accordingly, it is an excellent time to review the recent advances in tumor-targeting chemotherapy drugs by collecting articles from leading researchers at the 20th anniversary of the FDA approvals of these two epoch-making anticancer drugs with two different approaches to targeting cancer cells specifically. In this Special Issue, recent advances in these two approaches are presented.

Lyons *et al*.^[1]^ reviewed recent advances in preclinical research that have significantly increased the experimental rigor through which promising candidate molecules are accurately evaluated in parallel to the development of ADC technology. For example, preclinical tumor modeling with the development of patient-derived tumor organoid models that recapitulate many aspects of the human disease far better than conventional subcutaneous xenograft models. The authors also discussed how the application of several preclinical molecular imaging techniques has greatly enhanced the quality of experimental data, enabling quantitative pre- and post-treatment tumor measurements or the precise assessment of ADCs as effective diagnostics.

Venkatesh *et al*.^[2]^ presented an investigation into the redox properties of prostate-specific membrane antigen (PSMA) receptors in the ligand trafficking endosomes, which are crucial for the design and development of PSMA-targeted drug conjugates. Although there are different ways to implement drug release mechanisms, disulfide bond reduction has been extensively employed to ensure intracellular drug release in such ligand-based drug conjugates. Thus, the efficient receptor-targeted ligand-drug design must understand the redox properties of endocytic compartments. This study has revealed that PMSA-targeted ligand-drug probe is rapidly internalized by PSMA-positive cancer cells, but the release of the drug probe stops at ~50% even after 24 h. This means that not all internalizing receptors are translocated through similar intracellular compartments, and thus the efficiency of disulfide bond reduction must be independently analyzed for each receptor trafficking pathway.

It has been shown that RAS oncogenes are the most commonly mutated oncogenes in human cancer, while RAS-mutant cancers impose a heavy burden on human health. Although there have been some successes in the inhibition of RAS effector signaling, targeting these mutations is very challenging. Conroy *et al*.^[3]^ reviewed a newer approach, i.e., direct RAS inhibition, which has shown promising results in human clinical trials. In this review, diverse approaches to RAS inhibition are summarized, and then the recent successful developments in the direct inhibition of KRAS (G12C) with experimental drugs such as AMG510 (sotorasib), MRTX849 (Adagrasib), and BI-3406 are discussed.

Understanding the resistance mechanism is critically important in the discovery and development of anticancer agents targeting cancer-specific signaling pathways. Bijnsdorp and Peters^[4]^ investigated Rapamycin-resistance (mTOR signaling pathway) in human colon carcinoma cells and found that the drug resistance is caused by thymidine-induced autophagy activation. The authors examined if thymidine phosphorylase (TP) substrate thymidine and overexpression of TP affect mTOR signaling by comparing Colo320 (TP-deficient) cells and its TP-transfected variant (Colo320TP1). The results clearly showed that autophagy was highly induced by thymidine, which was decreased by the application of thymidine phosphorylase inhibitor. Furthermore, the autophagy inhibitor 3-methyladenine completely inhibited autophagy and its protection in Colo320TP1 cells.

The introduction of epidermal growth factor receptor (*EGFR*) gene tyrosine kinase inhibitors (TKIs) in chemonaive patients with advanced non-small cell lung cancer (NSCLC) has dramatically improved the progression-free survival compared to standard chemotherapy. However, the cost of treatment imposes a challenge in the wide use of these targeted chemotherapeutics as the first-line drugs in the clinic. Giuliani and Bonetti^[5] ^made a critical assessment of the pharmacological costs of TKIs (erlotinib, gefitinib, afatinib, and osimertinib) in patients with activating *EGFR *mutations in first-line treatment for advanced NSCLC. The authors concluded that osimertinib is most cost-effective based on incremental cost-effectiveness ratio, but still, reducing costs is mandatory to consider osimertinib as the most cost-effective TKI in first-line treatment.

In summary, this Special Issue includes review articles and original research articles, describing and addressing some critical aspects of the tumor-targeting chemotherapy drugs in preclinical and clinical studies.

## DECLARATIONS

### Copyright

© The Author(s) 2021.

## References

[1] Lyons SK, Plenker D, Trotman LC (2021). Advances in preclinical evaluation of experimental antibody-drug conjugates. Cancer Drug Resist.

[2] Venkatesh C, Shen J, Putt KS, Low PS (2021). Evaluation of the reducing potential of PSMA-containing endosomes by FRET imaging. Cancer Drug Resist.

[3] Conroy M, Cowzer D, Kolch W, Duffy AG (2021). Emerging RAS-directed therapies for cancer. Cancer Drug Resist.

[4] Bijnsdorp IV, Peters GJ (2021). Protective autophagy by thymidine causes resistance to rapamycin in colorectal cancer cells in vitro. Cancer Drug Resist.

[5] Giuliani J, Bonetti, A (2021). Cost-effectiveness of Osimertinib in activating epidermal growth factor receptor gene (EGFR)-mutations in first-line for advanced non-small cell lung cancer. Cancer Drug Resist.

